# *Panax notoginseng* Saponins Stimulates Neurogenesis and Neurological Restoration After Microsphere-Induced Cerebral Embolism in Rats Partially *Via* mTOR Signaling

**DOI:** 10.3389/fphar.2022.889404

**Published:** 2022-06-13

**Authors:** Jiale Gao, Jianxun Liu, Mingjiang Yao, Wei Zhang, Bin Yang, Guangrui Wang

**Affiliations:** ^1^ Beijing Key Laboratory of Pharmacology of Chinese Materia Medica, Institute of Basic Medical Sciences of Xiyuan Hospital, China Academy of Chinese Medical Sciences, Beijing, China; ^2^ Department of Pathology, Xiyuan Hospital, China Academy of Chinese Medical Sciences, Beijing, China

**Keywords:** *Panax notoginseng* saponins, cerebral ischemia, neurogenesis, synaptic plasticity, Akt/mTOR/p70S6K pathway

## Abstract

*P. Notoginseng* Saponins (PNS), the main active component of herbal medicine Panax notoginseng, has been widely used to treat cerebrovascular diseases. It has been acknowledged that PNS exerted protection on nerve injuries induced by ischemic stroke, however, the long-term impacts of PNS on the restoration of neurological defects and neuroregeneration after stroke have not been thoroughly studied and the underlying molecular mechanism of stimulating neurogenesis is difficult to precisely clarify, much more in-depth researches are badly needed. In the present study, cerebral ischemia injury was induced by microsphere embolism (ME) in rats. After 14 days, PNS administration relieved cerebral ischemia injury as evidenced by alleviating neurological deficits and reducing hippocampal pathological damage. What’s more, PNS stimulated hippocampal neurogenesis by promoting cell proliferation, migration and differentiation activity and modulated synaptic plasticity. Increased number of BrdU/Nestin, BrdU/DCX and NeuroD1-positive cells and upregulated synapse-related GAP43, SYP, and PSD95 expression were observed in the hippocampus. We hypothesized that upregulation of brain-derived neurotrophic factor (BDNF) expression and activation of Akt/mTOR/p70S6K signaling after ME could partially underlie the neuroprotective effects of PNS against cerebral ischemia injury. Our findings offer some new viewpoints into the beneficial roles of PNS against ischemic stroke.

## 1 Introduction

Ischemic stroke is one of the leading causes of long-term lethality and disability in the world (Zhu et al., 2018), and the incidence and prevalence are on the rise with an aging population globally (Koh and Park, 2017), which seriously threatens human health and brings heavy mental and economic burden to families and society (Liu et al., 2012). Stroke patients often suffer from sensory-motor and cognitive dysfunction, such as dementia, aphasia, paralysis, etc. However, ischemia stroke is involved in complex pathological mechanism and there is currently no effective preparation especially for neural restoration after stroke, so it is of great significance to develop drugs or preparations with neuroprotective and restorative effects in order to promote the recovery of neurological functions for stoke patients those who are out of 4.5-hour time window of thrombolysis.

In the past decade, much interest has been focused on neurogenesis, which provides fundamental support for remodeling and restoration of brain architecture and function (Yamaguchi et al., 2016) and may open up a novel therapeutic method to restore impaired neurological function after ischemic stroke. Nevertheless, neurogenesis from the embryonic brain throughout adulthood requires a well-controlled external and internal signal that guides the neural stem cells (NSCs) to transition into a properly functioning neuron, which involves the complex process of cell proliferation, division, differentiation, migration, and functional integration into neuronal circuits. In the adult brain, neurogenesis mainly happens in two canonical neurogenic areas: subgranular zone (SGZ) of dentate gyrus (DG) in the hippocampus and the subventricular zone (SVZ) of the lateral ventricle (Fh, 2000; Kempermann et al., 2015; Dillen et al., 2020), where endogenous NSCs proliferate, migrate and differentiate to replace dead neurons following cerebral ischemia (Kong et al., 2016; Piermartiri et al., 2020). However, due to limited repair capacity, ischemia-induced neurogenesis alone is not enough to restore neurological deficits, thus enhancing endogenous neurogenesis through drug stimulation might be an attractive strategy.

The main active ingredients of herbal medicine *P. notoginseng* are *P. notoginseng* saponins (PNS) whose major constituents are saponins, including Ginsenoside Rb1 (32.7 %), Ginsenoside Rg1 (32.1%), Notoginsenoside R1 (5.9%), Ginsenoside Rd (6.3%), and Ginsenoside Re (4.1%). It’s reported that PNS could modulate the inflammatory response and promote proliferation and differentiation of hippocampal NSCs *in vitro* (Si et al., 2011; Luo et al., 2021), indicating its potential benefits on neurogenesis after ischemic brain injury. Although the protection of PNS on neural damage induced by ischemic stroke is well characterized, the mechanism behind its actions concerning cerebral ischemia is not fully known. Further, the long-term effects of PNS on the recovery of neurological defects and neuroregeneration after stroke have not been thoroughly studied and the underlying molecular mechanism of stimulating neurogenesis is difficult to precisely clarify, much more in-depth researches are badly needed.

The mammalian target of rapamycin (mTOR), a large serine/threonine protein kinase, plays a vital role in cell growth, proliferation, survival, nutrient metabolism, autophagy, and protein translation, at present, great endeavor has been made to transfer targeting mTOR for cancer to CNS diseases and the extensive role of mTOR has attracted much interest in this field (Park et al., 2008; Guo and Yu, 2019). Multiple proteins combined with mTOR constitute two divergent complexes, designed as mTORC1 and mTORC2, in which mTORC1 is essential to maintain endogenous neural progenitor pool and for NSC differentiation into daughter cells (Licausi and Hartman, 2018). Studies have shown that AKT/mTOR signaling pathway could mediate the development of NSC, including NSC proliferation, differentiation, neural progenitor migration, dendrite development, synapse formation, and neuron maturation, playing a critical role in enhancing neurogenesis following ischemic stroke (Corsini et al., 2009; Liang et al., 2016; Zhang et al., 2016).

Regulation of cell signaling towards mTOR may exert profound effects on neurogenesis and initiate neuroprotection during cerebral ischemia, however, involvement of mTOR in cerebral ischemia-induced endogenous neurogenesis is not yet fully known, much more related researches are still needed to demonstrate this viewpoint. Here, we firstly demonstrated that PNS stimulated hippocampal neurogenesis by promoting NSC/NPC proliferation, migration, and differentiation activity and modulated synaptic plasticity by increasing synaptic formation-related proteins synthesis. Meanwhile, upregulation of brain-derived neurotrophic factor (BDNF) expression and activation of Akt/mTOR/p70S6K signaling after ME could partially underlie the neuroprotective effects of PNS against cerebral ischemia injury.

## 2 Materials and Methods

### 2.1 Main Chemicals and Reagents

PNS was offered by Chengdu Ruifen Si Biotechnology Co., Ltd. (Batch No. RFS-DFZY-SQZZG201225, Chengdu, China). CMC-Na was purchased from Dalian Meilun Biotechnology Co., Ltd. (Batch No. J1204A, Dalian, China). Sucrose was provided by Beijing Beihua Fine Chemicals Co., Ltd. (Batch No. 20051012, Beijing, China). Goat serum was purchased from Proteintech Group, Inc. (B900780, Wuhan, China). DAPI solution (C00650), 5′-Bromo-2′-deoxyuridine (BrdU, No. 2011031) and TritonX-100 (No. 1109F0524) were purchased from Solarbio Life Sciences & Technology Co., Ltd. (Beijing, China). GAP43 (ab75810), BDNF (ab108319), β-actin (ab8226-100), SYP (ab32127), PSD95 (ab238135), Anti-BrdU Rat mAb (ab6326), Anti-DCX Rabbit mAb (ab207175), Anti-NeuroD1 Rabbit mAb (ab213725), Anti-Nestin Rabbit mAb (ab221260) and Anti-Fade Fluorescence-aqueous, Fluoroshield (ab104135) were purchased from abcam (Cambridge, United Kingdom). Fluorescence microspheres (106–125 μm and 180–212 μm in diameter, UVPMS-BY2) were purchased from Cospheric (US). Cy3-labeled Goat Anti-Rat IgG (H&L) (CSA1041) and DyLight 488-labeled Goat Anti-Rabbit IgG (H&L) (CSA1029) were purchased from Cohesion (Cambridge, United Kingdom). Akt (4685s), Phospho-Akt (Ser473) (D9E) XP Rabbit mAb (p-Akt, 4060s), mTOR (2983), Phospho-mTOR (Ser2448) (D9C2) XP^®^ Rabbit mAb (p-mTOR, 5536), p70S6K (2708s), and Phospho-p70 S6 Kinase (Thr389) (D5U1O) Rabbit mAb (p-p70S6K, 97596s) were purchased from Cell Signaling Technology, Inc. (CST) (Boston, United States).

### 2.2 Experimental Animals

Male 8-week-old Sprague-Dawley (SD) rats (weighing 210 ± 10 g) were purchased from Beijing Sibafu Biotechnology Co., Ltd. [Beijing, China; laboratory animal certificate number: SYXK (jing), 2019-0010]. Animals were kept three to six per cage on a 12 h light/dark cycle with free access to water and food in a temperature (22°C ± 2°C) and humidity (55% ± 5%)-controlled Xiyuan hospital Animal Center. The experimental protocols were approved by Experimental Ethics Committee at Xiyuan hospital.

### 2.3 Animal Model and Drug Administration

#### 2.3.1 Experiment I

To evaluate the effect of PNS on the neurological protection of microsphere-induced cerebral embolism (ME) rats and screen out the effective doses of PNS, the rats were randomly divided into seven groups: sham, ME, ME + PNS 15 mg/kg, ME + PNS 30 mg/kg, ME + PNS 60 mg/kg, ME + PNS 120 mg/kg and ME + PNS 240 mg/kg groups (*n* = 9 per group). Microsphere-induced cerebral embolism (ME) was conducted to induce sustained cerebral ischemia according to Takeo et al., 1989 with slight modifications based on previous research in our laboratory.

In short, the rats were fastened in the supine position after their anesthesia by intraperitoneal injection of 80 mg/kg pentobarbital sodium. The right common carotid artery (CCA), internal carotid artery (ICA) and external carotid artery (ECA) were isolated and exposed, then the right pterygopalatine arteries were twisted off by electric coagulation pen and the right ECA were ligated with strings. Next, the right CCA for the rats were temporarily occluded with vascular clamp. Fluorescence microspheres, suspended in 200 ul of 5% dextran solution, were injected into the right ICA through a syringe inserted into the ECA, then the vascular clamp occluding CCA was simultaneously removed, allowing 273 microspheres to move to the various arteries of the brain and lead to embolisms, and the wound was closed by sutures. The sham rats received equal volume of vehicle without microspheres.

PNS was prepared fresh daily in 0.5% CMC-Na. The rats in PNS groups were administrated with 15, 30, 60, 120, or 240 mg/kg PNS accordingly once a day for 14 days *via* gavage after surgery. The rats in Sham and ME groups were given equal volume of vehicle *via* gavage. The fresh sterile solution of BrdU was made daily in 0.9% saline at a dilution of 10 mg/ml. For tracking the neurogenesis, all the rats were injected intraperitoneally with BrdU (50 mg/kg) once daily for 14 days in order to identify the proliferative cells. Fourteen days after ME, animals were killed by decapitation under anesthesia after the last BrdU injection.

In this part, we set five doses of PNS and explored the pharmacological protection effects of PNS on ME rats through animal behaviors, ELISA as well as HE staining experiments.

#### 2.3.2 Experiment II

Based on the results of Experiment I, we have selected three appropriately effective doses of PNS 30, 60, and 120 mg/kg to further investigate the effect of PNS on neurogenesis and synaptic plasticity, and finally identified the probable underlying mechanism through immunofluorescence and Western Blot experiments.

**FIGURE 1:**
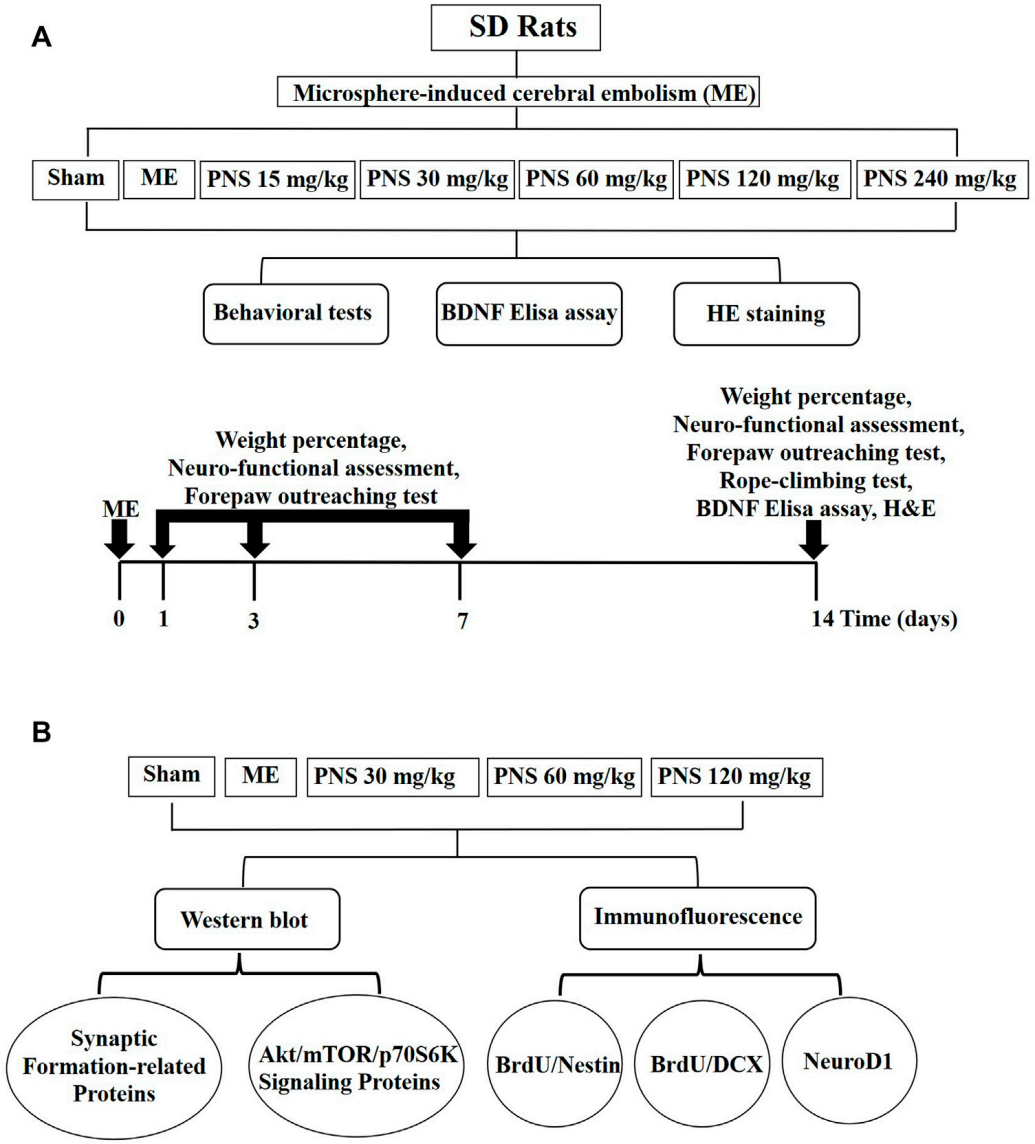
Diagram of experimental protocols. **(A)** Screening scheme of the appropriately effective PNS does **(B)** Mechanism of PNS promoting neural repair after cerebral ischemia.

The experimental protocols are presented in Figure 1.

### 2.4 Behavioral Test

The modified neuro-functional assessment, forepaw outreaching test and rope-climbing test were performed by a person blinded to group designation as previously described (Garcia et al., 1995; Ohlsson and Johansson, 1995; Wu et al., 2019b). Neuro-functional assessment and forepaw outreaching test were conducted at day 1, 3, 7, and 14 after operation, rope-climbing test were conducted at day 14 after operation.

#### 2.4.1 Weight Percentage

The body weight was measured every day until the end of experiment and the data were collected at day 1, 3, 7 and 14 after surgery for statistical analysis. Weight percentage = post-surgery body weight/pre-surgery body weight.

#### 2.4.2 Neuro-Functional Assessment

The neurological functional score was rated from 0 to 4 (0, no neurological deficit symptoms; 1, unable to completely stretch left forepaw; 2, circling to the left; 3, falling to the left or rolling on the ground; 4, no spontaneous activity with consciousness disorder). Rats with the score between one and three were included in the following experiments.

#### 2.4.3 Forepaw Outreaching Test

The rats were made to walk on forelimbs while being held by the tail. Symmetry in the outreaching of both forelimbs was observed with the hindlimbs of rats kept in the air, and the scores are as follows. 0, both forelimbs were outstretched symmetrically, and the rats walked in a straight line; 1, left forelimb outstretched less than right forelimb, and the rats leaned slightly to the left when walking; 2, left forelimb outstretched minimally, and the rats circled to the left when walking; 3, left forelimb did not move.

#### 2.4.4 Rope-Climbing Test

The rats were allowed to seize the steel rope (2 mm in diameter) 80 cm above the ground with their forelimbs while the hindlimbs were kept in the air and the time hanging on the steel rope was evaluated, the scores are the following: 0, seize the steel rope for more than 5 s with their hindlimbs tight to the rope; 1, seize the steel rope for 5 s without their hindlimbs tight to the rope; 2, seize the steel rope for 3∼4 s; 3, seize the steel rope for 0∼2 s.

### 2.5 Enzyme-Linked Immunosorbent Assay

The BDNF (brain derived neurotrophic factor) concentrations in the serum of rats after centrifugation (3000 rpm/min, 15 min) were measured using commercial ELISA kits (Human/Mouse/Rat Brain Derived Neurotrophic Factor Enzyme-Linked ImmunoSorbent Assay Kit, Beyotime Biotechnology) based on the manufacturer’s instructions.

### 2.6 Hematoxylin-Eosin Staining

Brains of rats in each group were separated and fixed using 10% formalin overnight, and were then dehydrated through graded alcohol and embedded in paraffin wax. Then paraffin-embedded tissue sections (5 μm thick) were stained routinely for HE staining in order to observe the neuronal pathological changes in the hippocampus.

### 2.7 Immunofluorescence

To explore the function of PNS on proliferation and migration of newly neural progenitor cells (NPC), expressions of BrdU/Nestin and BrdU/DCX in brain hippocampus were detected by double immunofluorescence staining. To study the impact of PNS on differentiation of neural progenitor cells, expressions of neurogenic differentiation1 (NeuroD1) in brain hippocampus were examined by immunofluorescence staining. Brains of rats were separated and fixed in 4% paraformaldehyde overnight at 4°C and then transferred into 25% sucrose in 0.1 M PB until sinking to the bottom. After that, the brains were embedded and frozen in optimal cutting temperature compound (OCT compound) and a series of brain coronal sections (40 μm) were cut at −20°C by a freezing microtome (Leica, CM1950, German). The frozen sections were then immediately processed for free-floating immunohistochemistry.

For BrdU/Nestin and BrdU/DCX double immunofluorescence staining, brain sections were washed in 0.1 M PB for 5 mins, then sections were incubated in 1 M HCl for 46 mins at 37°C to denature DNA, followed by 0.1 M sodium borate buffer (pH 8.5) for 10 mins at room temperature and rinsed three times for 5 mins each with 0.1 M PB. After that, brain sections were incubated in a blocking solution containing 3% normal goat serum and 1.5% TritonX-100 in 0.1 M PB for 30 min at room temperature prior to incubating at 4°C overnight in a solution containing 1% normal goat serum, 1.5% Triton X-100, with the primary antibodies [Rabbit anti-Nestin monoclonal antibody (1:100) or Rabbit anti-DCX monoclonal antibody (1:100)]. After rinsing three times for 5 mins each with 0.1 M PB, the sections were incubated 1.5 h at room temperature in the dark with corresponding secondary antibody [DyLight 488-labeled Goat Anti-Rabbit IgG (H&L) (1:400)]. Followed by rinsing in 0.1 M PB, brain sections were incubated in a blocking solution containing 3% normal goat serum and 1.5% TritonX-100 in 0.1 M PB prior to incubating in a solution containing 1% normal goat serum, 1.5% Triton X-100, with another primary antibody [Rat anti-BrdU monoclonal antibody (1:500)] at 37°C 1.5 h, and after rinsing three times for 5 mins each with 0.1 M PB, the sections were incubated 1.5 h at room temperature in the dark with corresponding secondary antibody [Cy3-labeled Goat Anti-Rat IgG (H&L) (1:500)]. DAPI solution was added for nuclear counterstaining.

For NeuroD1 immunofluorescence staining, brain sections were washed in 0.1 M PB for 5 mins, then sections were incubated in a blocking solution containing 3% normal goat serum and 1.5% TritonX-100 in 0.1 M PB for 30 min at room temperature prior to incubating at 4°C overnight in a solution containing 1% normal goat serum, 1.5% Triton X-100, with the primary antibody [Rabbit anti-NeuroD1 monoclonal antibody (1:200)]. After rinsing three times for 5 mins each with 0.1 M PB, the sections were incubated 1.5 h at room temperature in the dark with corresponding secondary antibody [DyLight 488-labeled Goat Anti-Rabbit IgG (H&L) (1:400)].

The images of immunofluorescence staining including NeuroD1 and double immunofluorescence staining including BrdU+/Nestin+ and BrdU+/DCX+ positive cells in ipsilateral subgranular zone (SGZ) and granule cell layer (GCL) of the hippocampal dentate gyrus were observed under the fluorescence microscope (Olympus, Olympus BX53, Japan) at a magnification of ×200. And the number of positive cells (Nestin/BrdU, DCX/BrdU and NeuroD1) was counted using ImageJ based on single-positive or double-positive cells and their nucleus in three coronal sections per animal (three animals in each group and three coronal sections per animal were used to separately determine the number of Nestin/BrdU, DCX/BrdU and NeuroD1-positive cells), which areas corresponded to coronal coordinates of −2.8 mm to −4.52 mm from bregma.

### 2.8 Western Blot

Fifteen rats were killed by decapitation (*n* = 3 per group) and their brains were rapidly taken and then frozen by liquid nitrogen. The ipsilateral hippocampus was isolated and homogenized in RIPA lysis solution containing phosphatase inhibitors, protein phosphatase inhibitors and PMSF on ice for thorough lysis of proteins, then the supernatant was gathered by centrifugation. Bradford method was adopted to detect the protein contents and then protein concentration was adjusted to the same level after quantification. Then it was mixed with 5× loading buffer to prepare sample solutions of a certain concentration and samples were separated by SDS-PAGE, and then transferred to PVDF membranes (Millipore, United States). Membranes were blocked with 5% non-fatty milk or 5% BSA for 2 h, followed by incubation overnight at 4°C with the primary antibodies [Akt (1:1000), p-Akt (1:1000), mTOR (1:1000), p-mTOR (1:1000), p70S6K (1:1000), p-p70S6K (1:500), GAP43 (1:1000), SYP(1:1000), PSD95 (1:1000), BDNF (1:1000) and β-actin (1:1000)]. Then, the membranes were washed with TBST for 5 min three times, and were incubated for another 1 h at room temperature with corresponding secondary antibodies. Immunoreactive bands were detected using BeyoECL Moon reagent (beyotime, China) and the average gray value of each band was calculated using the software ImageJ, β-actin was used as an internal control.

### 2.9 Statistical Analysis

All data were presented as mean ± standard deviation and were analyzed using appropriate statistical methods with SPSS 25.0 software (IBM, Chicago, United States). Differences between two groups were evaluated by a non-parametric *U*-test when data were out of normal distribution. Statistical comparison among multiple groups was determined through a one-way analysis of variance or two-way repeated-measures analysis of variance followed by the LSD test, and *p* < 0.05 indicates a significant difference.

## 3 Results

### 3.1 *P. notoginseng* Saponins Treatment Improved the Recovery of Neurological Functions in Microsphere Embolism Rats

**FIGURE 2:**
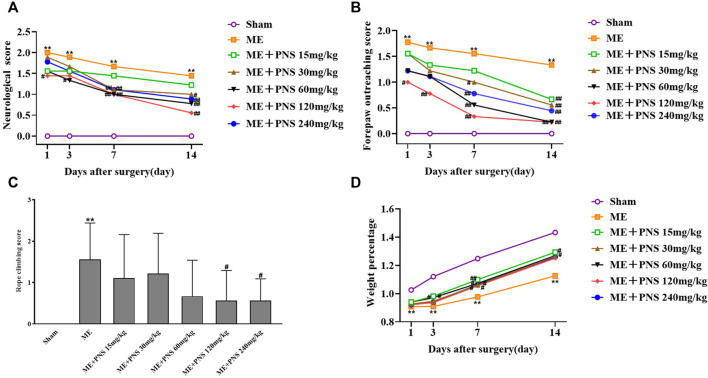
PNS treatment improved the recovery of neurological functions in ME rats. Comparison of neurological score, forepaw outreaching score and weight percentage were measured at day 1, 3, 7, and 14 after ME surgery at different time nodes. Rope climbing test score were detected at day 14 after ME surgery. **(A)** Neurological score **(B)** Forepaw outreaching test score **(C)** Rope climbing test score **(D)** Weight percentage. The data in experiment **(A,B,D)** were shown as means and the data in experiment **(C)** were presented as means ± standard deviation (*n* = 9 animals per group). *p < 0.05, **p < 0.01 versus Sham group; ^#^ *p* < 0.05, ^##^ *p* < 0.01 versus ME group.

As shown in Figures 2A–C, the effect of PNS on ME-induced neurological defects was evaluated by neurological score, forepaw outreaching and rope-climbing test score. The higher the scores were, the severer the damage was. The neurological score, forepaw outreaching test score as well as rope-climbing test score were all strikingly increased in ME group compared with sham group at different time nodes, indicating a severe neurological impairment. However, both the neurological score and forepaw outreaching score of ME rats obviously decreased over time after surgery, which means mild recovery after ME, and PNS administration effectively attenuated the neurological deficits in ME rats at different time nodes. Compared with ME group, on day 1 after surgery, PNS 120 mg/kg significantly reduced the neurological score and forepaw outreaching score, on day 3 after surgery, PNS 120 mg/kg significantly reduced the forepaw outreaching score and PNS 60 mg/kg significantly reduced neurological score, on day 7 after surgery, PNS 30, 60, 120, and 240 mg/kg significantly reduced the neurological score and forepaw outreaching score, on day 14 after surgery, PNS 30, 60, 120, and 240 mg/kg significantly reduced the neurological score and forepaw outreaching score, PNS 15 mg/kg significantly reduced the forepaw outreaching score, and PNS 120 and 240 mg/kg significantly reduced the rope-climbing test score. Overall, PNS 30, 60, and 120 mg/kg group appeared to function relatively well. As shown in Figure 2D, remarkedly time-dependent increase in weight percentage of Sham, ME and PNS treatment rats were all observed, and the weight percentage was obviously decreased in ME group compared with sham group at different time nodes, however, treatment with PNS treatment exhibited an increase in ME rats on day 3, 7, and 14 after surgery. Compared with ME group, on day 3 after surgery, PNS 15 and 30 mg/kg significantly increased the weight percentage, on day 7 after surgery, PNS 15, 30, 60, 120, and 240 mg/kg significantly increased the weight percentage, and on day 14 after surgery, PNS 15 and 60 mg/kg significantly increased the weight percentage. Taken together, these results indicated that PNS treatment ameliorated neurological function deficits induced by ME in rats.

### 3.2 *P. notoginseng* Saponins Administration Attenuated Microsphere Embolism-Induced Decrease of Brain-Derived Neurotrophic Factor Content in the Serum

**FIGURE 3:**
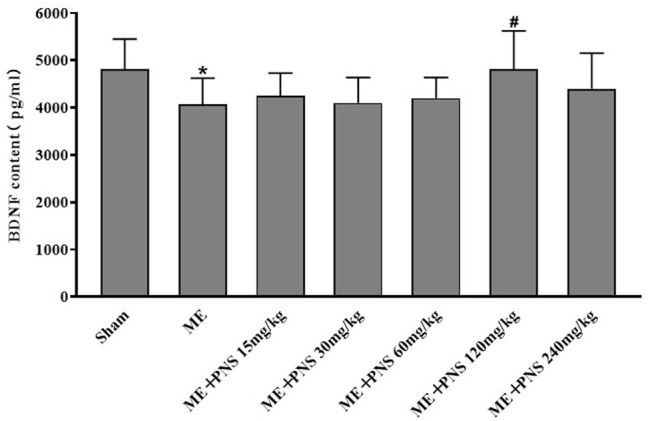
PNS administration promoted BDNF protein levels in the serum of ME rats. Expression level of BDNF was measured by ELISA and data were presented as means ± standard deviation, n = 8 in sham and ME, n = 7 in other groups. *p < 0.05, **p < 0.01 versus Sham group; ^#^ *p* < 0.05, ^##^ *p* < 0.01 versus ME group.

As shown in Figure 3, ME-induced cerebral ischemia led to an obvious downregulation of BDNF protein level in the serum compared with sham group, whereas administration with 15, 30, 60, 120, and 240 mg/kg PNS for 14 days all attenuated ME-induced decrease of BDNF content. Among which, ME + PNS 120 mg/kg group significantly increased the BDNF concentration after ME.

### 3.3 *P. notoginseng* Saponins Administration Increased the Number of Viable Neurons and Alleviated Pathological Damage in Hippocampus of Microsphere Embolism Rats

**FIGURE 4:**
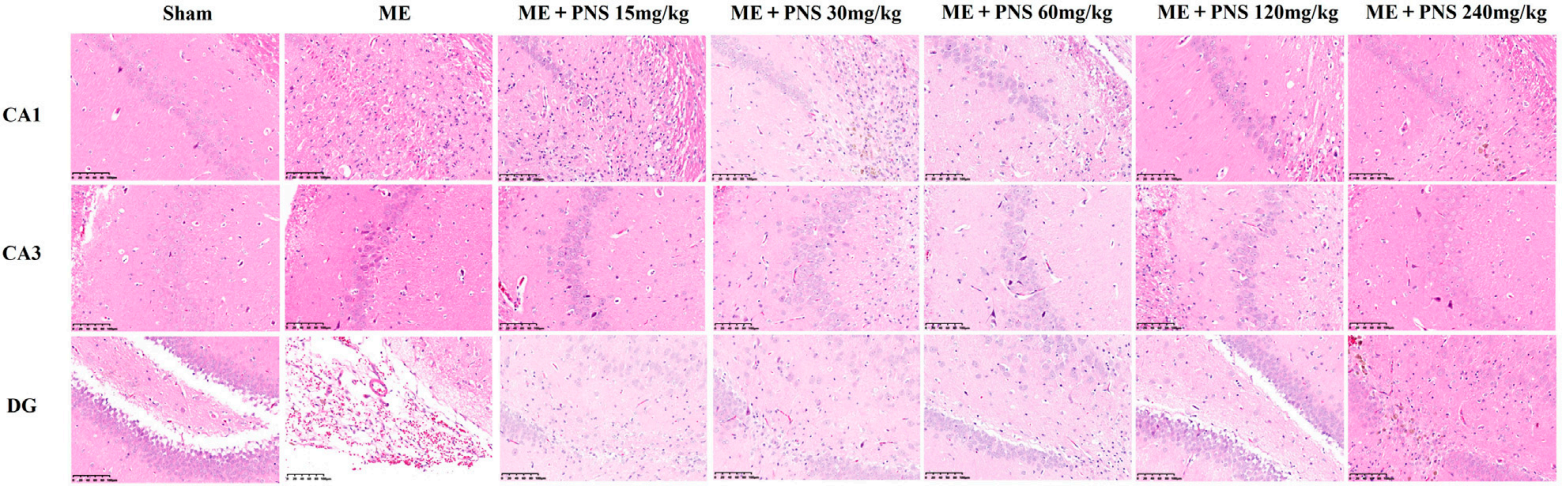
Effect of PNS on histopathological changes of hippocampal CA1, CA3, and DG in ME rats under a ×200 light microscope (scale bar = 100 μm).

HE staining was carried out to investigate the morphological changes of brain tissue. As shown in Figure 4, neuronal cells in the CA1, CA3, and DG regions of hippocampus were intact without vacuoles and necrosis, the cytoplasm was rich and uniform, cells were arranged neatly and connected tightly in sham group. But in ME group, obvious neuronal injuries were observed, including infarctions and a large area of vacuoles, accompanied by inflammatory cell infiltration. The neuronal cells were loosed and disordered, the widened intercellular space and decreased intact neurons could be clearly seen. However, these histopathological injuries were attenuated after PNS treatment and smaller infarctions, reduced inflammatory cell infiltration and more intact neurons were found in PNS-treated groups, among which ME + PNS 30, 60, or 120 mg/kg group seemed to play a relatively good effect.

These above experimental results showed that PNS 30, 60, or 120 mg/kg presented the optimal effect, may be the appropriate doses and were then enrolled in the following experiments to further explore the effect of PNS on neurogenesis, synaptic plasticity and underlying mechanism through immunofluorescence and Western Blot.

### 3.4 *P. notoginseng* Saponins Administration Stimulated Post-ischemic Hippocampal Neurogenesis in Microsphere Embolism Rats

To determine whether the long-term effect of PNS on ischemic stroke benefits from the proliferation, migration and differentiation of newborn neuronal cells, we detected the BrdU/Nestin, BrdU/DCX double labelled and NeuroD1 single labelled, presumably NSC/NPCs, migrating neuroblasts and differentiated immature neurons respectively.

**FIGURE 5:**
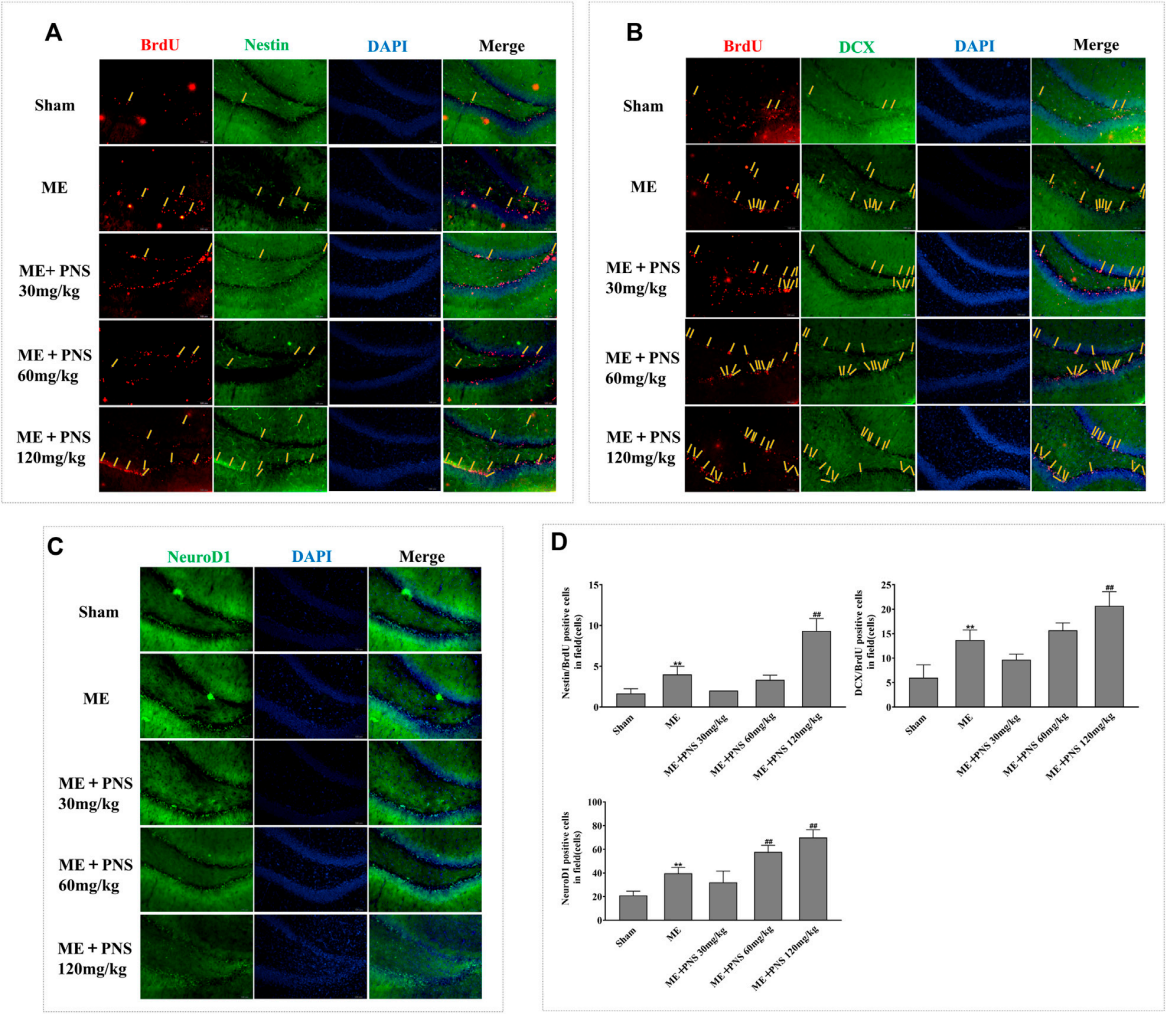
PNS administration stimulated post-ischemic hippocampal neurogenesis in ME rats. Representative images of ipsilateral hemisphere sctions under a fluorescence microscope at a magnification of ×200 (scale bar =100 μm) were shown as **(A)** BrdU (red, a marker of proliferating cells) and Nestin (green, a marker of NSC/NPC); **(B)** BrdU (red) and DCX (green, a marker of migrating neuroblasts); **(C)** NeuroD1 (green, a marker of differentiation factor). **(D)** The numbers of double-positive of BrdU/Nestin, BrdU/DCX, and single-positive NeuroD1 were analyzed and data were presented as means ± standard deviation (n = 3 animals per group). *p < 0.05, **p < 0.01 versus Sham group; ^#^ *p* < 0.05, ^##^ *p* < 0.01 versus ME group.

As illustrated in Figures 5A,B,D, in sham group, almost few BrdU/Nestin and BrdU/DCX were found in DG, however, both the numbers of BrdU/Nestin and BrdU/DCX were remarkably increased in damaged DG zone of ME rats, demonstrating that the proliferation and migration of NSC/NPC were triggered in response to cerebral ischemia. Besides, both the double-positive cells of BrdU/Nestin and BrdU/DCX in PNS 120 mg/kg group were significantly upregulated compared with ME rats, showing that PNS owned the ability to promote NSC/NPC proliferation and migration after cerebral ischemia.

Similarly, as we can see from Figures 5C,D, in the impaired DG of ME rats, NeuroD1 obviously expanded as compared with sham group. Besides, the number of NeuroD1-positive cell of PNS 60 and 120 mg/kg group was obviously higher than that of ME group, showing that PNS could further promote the neuronal differentiation.

These findings suggested that PNS could enhance the proliferation, migration and differentiation of NSC/NPC in the DG after ME.

### 3.5 *P. notoginseng* Saponins Administration Modulated Synaptic Plasticity by Increasing the Expressions of Brain-Derived Neurotrophic Factor and Synaptic Formation-Related Proteins GAP43, SYP, and PSD95 in the Hippocampus of Microsphere Embolism Rats

**FIGURE 6:**
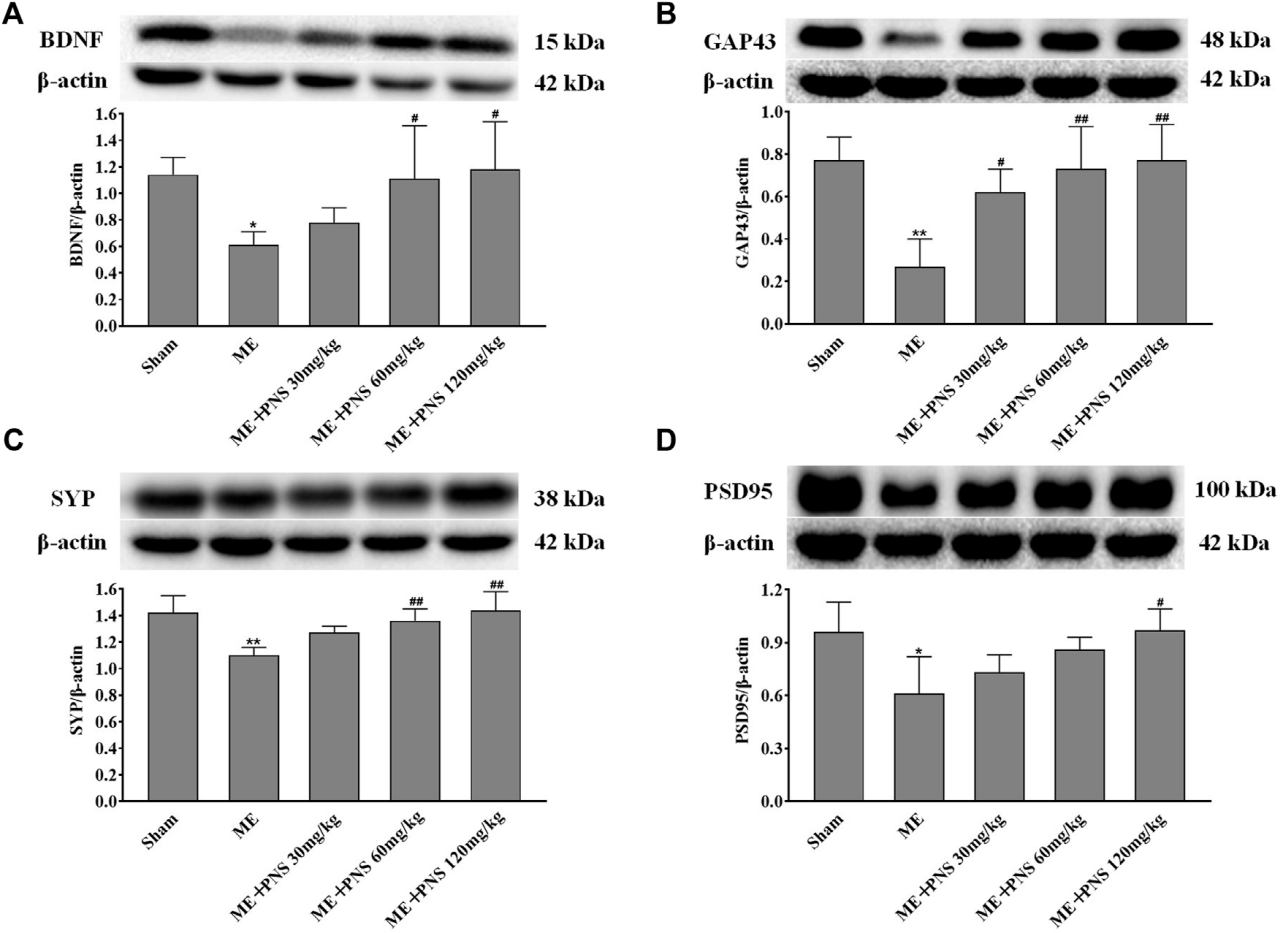
PNS administration enhanced synaptic plasticity by increasing the expressions of BDNF and synaptic formation-related proteins GAP43, SYP, and PSD95 in hippocampus of ME Rats. The expressions of BDNF **(A)**, GAP43 **(B)**, SYP **(C)**, and PSD95 **(D)** in hippocampus tissues were assessed by Western blot. Data were presented as means ± standard deviation (*n* = 3 animals per group). *p < 0.05, **p < 0.01 versus Sham group; ^#^ *p* < 0.05, ^##^ *p* < 0.01 versus ME group.

BDNF, a key neurotrophic factor produced in the brain, has been proved to protect neurons and promote neurogenesis and synaptic plasticity during ischemic stroke. To evaluate the effects of PNS on synaptic connectivity after cerebral ischemia, BDNF, GAP43 (the crucial component of axonal outgrowth), SYP (presynaptic marker), PSD95 (postsynaptic marker) were examined. As presented in Figures 6A–D, the expression levels of BDNF, GAP43, SYP, and PSD95 were all significantly downregulated in response to ME, which was reversely promoted by PNS administration in a dose-dependent manner, indicating that PNS was possibly involved in the beneficial effect of synaptic plasticity in ME rats.

### 3.6 *P. notoginseng* Saponins Administration Activated Akt/mTOR/p70S6K Pathway in Microsphere Embolism Rats

**FIGURE 7:**
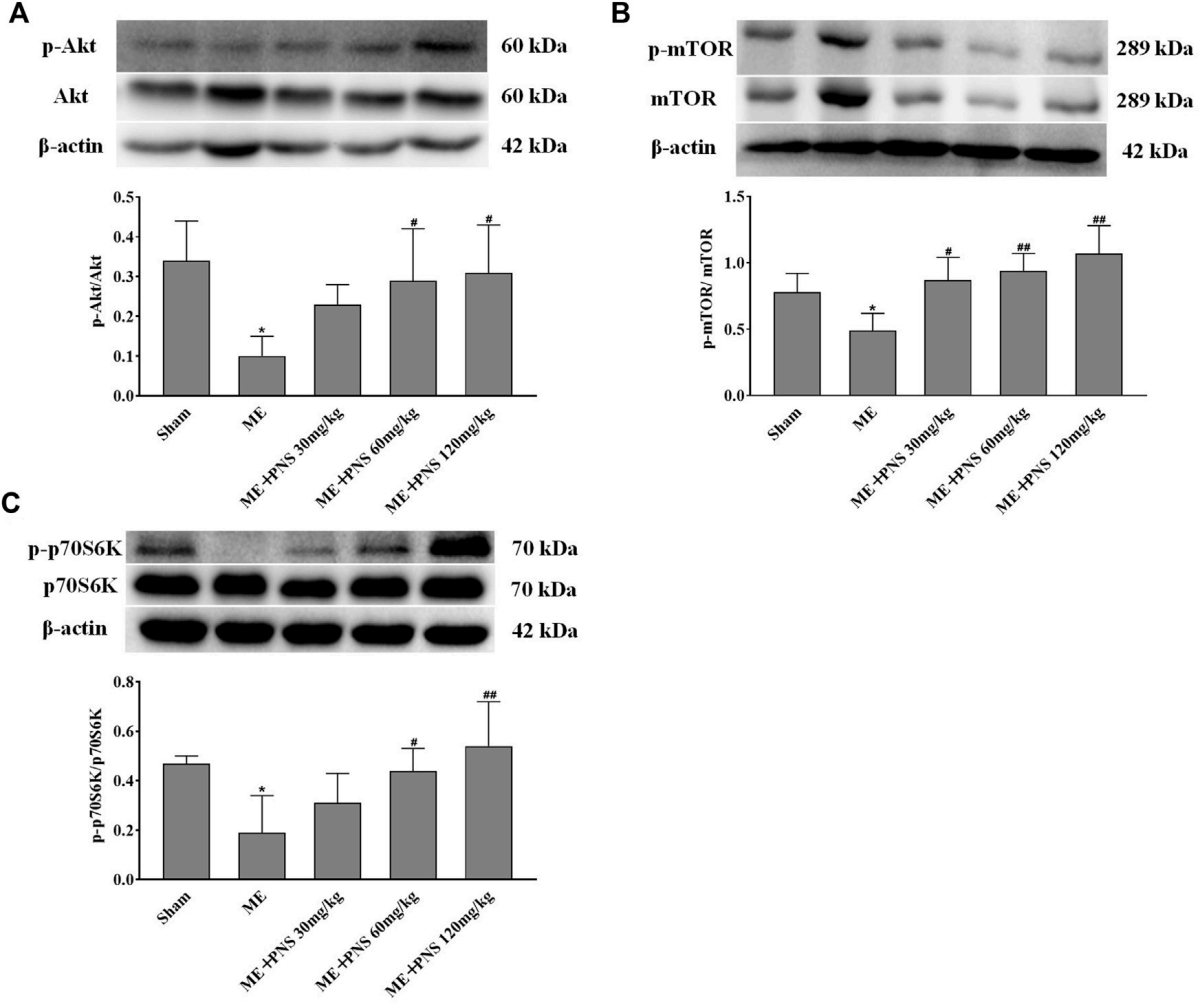
PNS administration activated Akt/mTOR/p70S6K pathway in ME rats. (**A−C**) The protein levels of p-Akt, Akt, p-mTOR, mTOR, p-p70S6K, and p70S6K in hippocampus tissues were detected by Western blot. Data were shown as means ± standard deviation (*n* = 3 animals per group). *p < 0.05, **p < 0.01 verssus Sham group; ^#^ *p* < 0.05, ^##^ *p* < 0.01 versus ME group.

AKT/mTOR signaling plays a crucial role in stimulating neurogenesis and synaptic plasticity. To further explore the potential molecular mechanisms of PNS in promoting neurogenesis and expression of synaptic formation-related proteins induced by ME, we investigated whether PNS results in some changes in the AKT-mTOR-p70S6K pathway. The protein levels of p-Akt, Akt, p-mTOR, mTOR, p-p70S6K, and p70S6K were measured by Western blot. As depicted in Figures 7A–C, the ratios of p-Akt/Akt, p-mTOR/mTOR, and p-p70S6K/p70S6K remarkably descended after ME. However, PNS administration dramatically raised the p-Akt/Akt, p-mTOR/mTOR, and p-p70S6K/p70S6K ratios in a dose-dependent manner, suggesting that Akt/mTOR/p70S6K pathway might participate in the protective mechanisms of PNS against cerebral ischemia injury.

## 4 Discussion

Stroke is a leading cause of physical disability and death worldwide, characterized by high rates of morbidity, mortality, disability and recurrence, including hemorrhagic and ischemic stroke, and the latter accounts for a large proportion (Liu et al., 2019; Sun et al., 2020b). Although there are many drugs developed to cure stroke symptoms, the effective treatment for management in patients is limited and sequelae of severe neurological damage remain irreversible. Ischemia-induced spontaneous neurogenesis is beneficial but not sufficient, therefore in the present study, we explored the effect of PNS on endogenous NSCs/NPCs in a rat of ME model.

Oral clinical preparations with PNS as the main component, such as Xueshuantong soft capsule, Xuesaitong tablet or granule, were often used to treat cerebrovascular sequelae, and the clinical PNS dosage was 2.5–10 mg/kg individually per day, which could be transformed into 15.75–63 mg/kg daily for rats (obversion coefficient = 6.3). What’s more, plenty of previous studies have shown that 30–120 mg/kg PNS is optimal for protection against cerebral ischemia injuries in animal models (Wu et al., 2016), and lower or higher dose, such as 10 or 300 mg/kg, has also been orally given to the rats in some studies (Fan et al., 2015; Zhou et al., 2018). Therefore, in this study, PNS dosage was selected as 15, 30, 60, 120, and 240 mg/kg, which is 1.5, 3, 6, 12, and 24 times the maximum clinical dose (10 mg/kg) respectively. Our results showed that PNS treatment ameliorated neurological function deficits induced by ME in rats, and PNS 30, 60 and 120 mg/kg seemed to play a relatively good effect.

Our findings revealed that PNS relieved neurological deficits and hippocampal pathological damage caused by cerebral ischemia. Moreover, PNS increased hippocampal neurogenesis by reinforcing the proliferation, migration and differentiation activity of NSC/NPCs in the post-ischemic DG and modulated synaptic plasticity by strengthening the expression of synaptic formation-related proteins GAP43, SYP, and PSD95 in the hippocampus. Meanwhile, BDNF upregulation and activation of Akt/mTOR/p70S6K pathway could partially underlie the neuroprotective effects of PNS against cerebral ischemia injury.

Cerebral ischemia could stimulate neurogenesis in the DG and enhanced hippocampal neurogenesis may be a compensatory adaptive response to ischemia-induced injuries, which contributes to improvement of neurological function and remodeling of brain structures after stroke (Liu et al., 1998; Sun et al., 2020a). Enhancing endogenous neurogenesis is important for recovery of neurological function after stroke, and some studies have shown that neurological deficits were improved in ischemic stroke rat *via* increasing neurogenesis (Li et al., 2018; Cheng et al., 2020). Our results showed that PNS alleviated neurological deficits and hippocampal pathological damage after ischemic stroke, then to determine whether improved neurological function benefited from enhanced neurogenesis in hippocampal dentate gyrus, we detected major indicators of neurogenesis by immunofluorescence. In the DG, newly born neurons are locally generated at the border between the hilus and the granule cell layer and then migrate into the granule cell layer, where they gradually matured both morphologically and functionally and finally integrated into neural circuits (Stanfield and Trice, 1988). Here, we used BrdU (5-Bromo-2-deoxyUridine) as a principal proliferative marker of only dividing cells during neurogenesis, which is an analogue for an endogenous DNA base thymidine, could track the fate of divided cells and their progeny *via* the substitution of thymidine during the S phase of mitosis and participate in the process of newly synthesized DNA (Moon et al., 2016). As an initial response to neurogenesis, it is necessary to enhance endogenous NSC proliferation, which helps to promote the migration, differentiation and survival of newborn neurons. Nestin has been widely recognized as a marker of NSC/NPCs and double-labelled BrdU/Nestin was used for the identification of newly generated NSCs in the DG (von Bohlen und Halbach, 2011; Zhang et al., 2020). Therefore, we used BrdU/Nestin to evaluate DG cellular proliferative ability at day 14 post-ME, and the immunofluorescence staining results presented that BrdU/Nestin expression in the DG began to increase after ME, which denoted an activated proliferative characteristic of NSC as a response to cerebral ischemia, and PNS 120 mg/kg administration remarkedly advanced BrdU/Nestin expression, suggesting that PNS could augment the number of NSC populations in the hippocampus. Doublecortin (DCX) is a microtubule-associated protein, to better specify whether PNS have potential benefits on immature neuroblasts, we detected BrdU/DCX double-labelled cell, which is usually accepted as a marker of newborn migrating neuroblasts (Gleeson et al., 1999). Researchers reported that PNS treatment enhanced DCX^+^ expressions in the olfactory bulb at day 14 after global brain ischemia/reperfusion (He et al., 2015). Similar to these results, our findings revealed that expressions of BrdU/DCX in the DG of PNS 60 and 120 mg/kg groups were higher than ME groups, showing that PNS boosted NSCs to differentiate into immature neuroblasts. NeuroD1, a bHLH transcription factor indispensable for granule neuron differentiation, could foster the precursor cell lineage into immature neuron through differentiation (Hodge and Hevner, 2011; Brulet et al., 2017). In accordance with previous studies (Kisoh et al., 2017), our results also exhibited significantly upregulated expression of NeuroD1-positive cells in the DG after ME, which was further strengthened by PNS 60 and 120 mg/kg administration. All these results demonstrated that PNS advanced neurogenesis by reinforcing the proliferation, migration and differentiation ability of newborn cells after ME. And we speculated that enhancing the proliferation, migration, and differentiation of NSC/NPC after ME by PNS administration might be involved in the recovery of neurological function.

BDNF is regarded as an instructive intermedia of functional and structural plasticity in the brain, playing a critical role in enhancing adult hippocampal neurogenesis (Liu et al., 2018; Colucci-D’Amato et al., 2020). And low BDNF concentration has been associated with the rising risk of stroke. Researchers found that patients with acute stroke had significantly lower BDNF levels in the serum compared to healthy controls and decreased concentration of BDNF in the serum was also found in cerebral ischemic rat model (Chen et al., 2012; Karantali et al., 2021). Moreover, previous studies have shown that levels of BDNF in the hippocampus were found to be obviously lower in ischemic rats than sham rats (Moriyama et al., 2011; Sheikholeslami et al., 2021). Therefore, in this study, we detected the levels of BDNF both in the serum and hippocampus tissue, consistent with these observations, our studies showed that BDNF levels in the serum and hippocampus of ME rats were both obviously decreased compared with sham rats, however, administration with 15, 30, 60, 120, and 240 mg/kg PNS for 14 days all attenuated ME-induced decrease of BDNF content in the serum. Among which, PNS 120 mg/kg group remarkedly increased the BDNF concentration in the serum after ME surgery. And BDNF expression in the hippocampus of PNS 30, 60, and 120 mg/kg groups was higher than ME group. We speculated that BDNF induction may be related to the protective mechanisms of PNS against cerebral ischemia injury.

Activating the mTOR signaling pathway revitalizes the NSCs, restores their proliferation and enhances hippocampal neurogenesis (Romine et al., 2015). The activity of mTOR is regulated through phosphorylation on its specific residue serine 2448, which is the target of upstream Akt and downstream p70 ribosomal S6 kinase (p70S6K), and phosphorylation of threonine 389 residue by mTOR is critical for p70S6K activation and serves as a marker for mTOR activity (Chong et al., 2010; Chong et al., 2013). When AKT/mTOR pathway is activated, the phosphorylation level of downstream substrate p70S6K is remarkably upregulated, thus the development, differentiation, survival and regeneration of neurons as well as protein translation can be promoted (Li et al., 2020).To further explore potential mechanisms of enhanced neurogenesis in the hippocampal DG after PNS treatment, we next chose hippocampus tissue to examine the expression of Akt-mTOR-p70S6K signaling. Our western blot results have shown that the ratio of p-Akt/Akt, p-mTOR/mTOR, and p-p70S6K/p70S6K was obviously reduced after ME compared with sham group, however, PNS administration enhanced phosphorylation of Akt, mTOR, and p70S6K dose-dependently after ME. We supposed that these quantitative changes of Akt, mTOR and p70S6K were caused by NSC in the hippocampal DG, and induction of neurogenesis was possibly associated with activation of Akt/mTOR/p70S6K signaling pathway.

After cerebral ischemia, axon remodeling and synaptic connectivity are critical for neurorehabilitation. Differentiated mature neurons are highly polarized cells that own two units, namely axons and dendrites, and transmission information between axons and dendrites in neurons mainly depends on synaptic function and plasticity. As a principal modulator of translation, activation of mTOR has also been linked with synapse-related protein synthesis and synaptic plasticity (Li et al., 2010; Xie et al., 2018; Xiong et al., 2018). Not only does BDNF have the ability to promote neurogenesis, but also appears to be vital to synaptic function and plasticity in the adult hippocampus (Rauti et al., 2020). Having proved that PNS increased BDNF expression and activated the Akt/mTOR/p70S6K pathway after ME in this study, we then focused on the expression of synapse-related proteins in the hippocampus to further identify whether PNS could also restore the disrupted synaptic function after cerebral ischemia. GAP43 (growth associated protein 43), a crucial component of axonal outgrowth, is involved in neurite outgrowth and axon regeneration during neuronal development (Abe et al., 2010; Yang et al., 2020). Previous studies found that GAP43 expression was obviously decreased after cerebral ischemia/reperfusion insults (Zhang et al., 2019; Chen et al., 2020) and similar to these results, GAP43 level was also dramatically declined after ME in our study, whereas PNS administration enhanced axonal growth capacity by upregulating GAP43 expression with a dose-dependent trend. The presynaptic marker SYP (synaptophysin) and postsynaptic marker PSD95 (post-synaptic density protein 95) are two major synaptic proteins, which were associated closely with synaptic formation and neurotransmission (Wu et al., 2019a). The nerve terminal of neurons is filled with some small synaptic vesicles, specialized secretory organelles participated in the storage and release of neurotransmitters, and SYP (presynaptic marker) is the major integral membrane protein of synaptic vesicles, indicates connections between neurons (Thiel, 1993; Cousin, 2021). PSD95 (postsynaptic scaffold protein) has been proved to be required for the final stages of morphological maturation and formation of dendritic spines, which are vital for the integration of hippocampal granule neurons (Mardones et al., 2019). Our results found that the expression of SYP and PSD95 proteins profoundly decreased after ME, which was in agreement with previous study showing downregulation of synaptic proteins after ischemia in adult animals (Zhang et al., 2018), indicating a failure in synaptic functioning. However, rats treated with PNS showed a higher expression of SYP and PSD95 than ME rats. We speculated the upregulation of GAP43, SYP, and PSD95 in the hippocampus by PNS administration were originated from nerve cells, mainly from neurons, implying that PNS probably induced production of synaptic connectivity between neurons. Overall, the above results revealed that PNS may contribute to mediating hippocampal synaptic plasticity through invoking the synapse-related proteins expression of GAP43, SYP, and PSD95.

Enhancing endogenous neurogenesis through drug stimulation might be an attractive strategy to recover the damaged neurons after stroke, and the ability of PNS to synchronously offer neuroprotection, promote neurogenesis and modulate synaptic plasticity perhaps make it a promising candidate for developing strategies to stimulate NSC/NPCs for neural repair after cerebral ischemia. In spite of these encouraging findings, whether these newborn NSC/NPC could gradually develop into mature neurons both structurally and functionally, forming appropriate synapse between newly NPC-derived neurons and host neurons and finally integrating into existing neural circuits needs to be explored in-depth in the future.

## 5 Conclusion

Taken together, our study demonstrated that PNS administration enhanced the recovery of neurological deficits and improved hippocampal pathological damage caused by cerebral ischemia. What’s more, PNS stimulated hippocampal neurogenesis by promoting NSC/NPC proliferation, migration and differentiation activity and modulated synaptic plasticity. Meanwhile, upregulation of BDNF expression and activation of Akt/mTOR/p70S6K signaling after ME could partially underlie the neuroprotective effects of PNS against cerebral ischemia injury. Our findings offer some new standpoints into the beneficial roles of PNS against ischemic stroke.

## Data Availability

The original contributions presented in the study are included in the article/Supplementary Materials, further inquiries can be directed to the corresponding author.
